# Do ostensive verbal signals have a unique importance when communicating with dogs?

**DOI:** 10.1017/ehs.2025.10031

**Published:** 2025-12-02

**Authors:** Petra Dobos, Csenge Anna Lugosi, Péter Pongrácz

**Affiliations:** Department of Ethology, ELTE Eötvös Loránd University, Budapest, Hungary

**Keywords:** ostensive speech, neutral intonation, dog–human interactions, functional breed selection, perseveration

## Abstract

Just like in human infants, ostensive verbal utterances can transform human actions into a natural teaching scenario for dogs. However, functional selection created ‘independent’ and ‘cooperative’ dog breeds with different dependence on human signals. We hypothesize that this could affect dogs’ sensitivity towards verbal communication. We tested independent and cooperative breeds in the two-choice ‘A-not-B paradigm’. The experimenter used either ostensive or neutral intonation speech while hiding the target. Based on the target’s position, the trial order was A-A-B-B-A. Perseverative ‘A-not-B’ errors in Trial 3 are interpreted as learning the rule to look for the reward at location ‘A’. From the near 100% success rate in Trials 1 and 2, each groups’ performance dropped to chance level in Trial 3, except for cooperative dogs in the neutral speech condition. Independent dogs in the neutral speech condition paid the least attention to the experimenter. We conclude that perseverative errors can be either the consequence of rule-learning elicited by ostensive intonation or reverting to the ‘win–stay’ strategy, when independent dogs lost interest in watching where the experimenter exactly hid the reward. Functional selection could influence dogs’ general attentiveness towards human communication; thus, neutral speech may have an underestimated relevance for cooperative dogs.

## Graphical abstract

**Figure:**
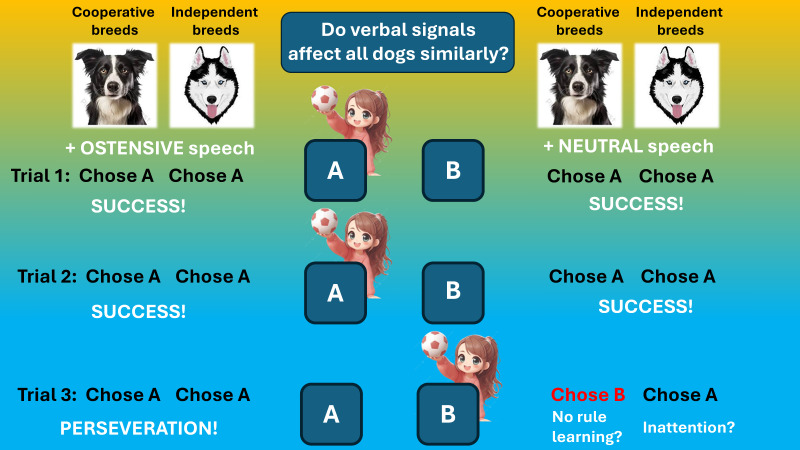

## Social media summary

Dogs who were bred to cooperate with us will pay attention, whatever we say to them with any kind of intonation.

## Introduction

Complex social cognition is probably the most important intrinsic factor that enables and maintains the interactions and relationships among individuals of highly social vertebrate species (Seyfarth & Cheney, 2015; Zuberbühler & Byrne, 2006). Domesticated animals had to adapt to a new, anthropogenic environment (Zeder, 2015), where effective (conflict-minimizing and amicable) human–animal interactions could be aided by the ‘exaptation’ of already existing (Hansen-Wheat et al., 2018; Hansen-Wheat & Temrin, 2020) or developing novel sociocognitive traits (Miklósi & Topál, 2013). From among these traits, communicative capacities that helped domesticated animals to interact with people both as senders (Greenall et al., 2022; Pongrácz, 2017) and receivers (e.g., Boros et al., 2024) of signals during interspecific communication are considered of high hypothetical value (Kleinau et al., 2024).

Although the role of life histories (Udell et al., 2008), environment (Zaine et al., 2015), and learning (Marshall-Pescini *et al*., 2016) cannot be underestimated, there is compelling evidence that the dog as a species possesses the capacity for developing highly successful social understanding towards human communication (Virányi et al., 2008) as they engage in cooperative actions with them (Range & Virányi, 2015) and initiate communication and joint activities with humans (Miklósi et al., 2003). These sociocognitive features could provide dogs with an advantage in their adaptation to the anthropogenic niche (e.g., bonding with humans:Boitani et al., 2007; choosing of their resting place: Sen Majumder et al., 2016; interacting with humans: Bhattacharjee et al., 2017). The sociocognitive features of dogs could also serve as further basis in directional artificial selection for various tasks alongside humans (Hare & Ferrans, 2021).

It is widely accepted that the most fundamental sociocognitive capacities that have helped dogs to become a uniquely successful working aid and companion to humans (Asp et al., 2015; Pongrácz & Dobos, 2023) are likely present in all dogs, including free-ranging dogs (Sarkar & Bhadra, 2022). At the same time, it remains an intriguing question whether the well-documented between-individual differences in various sociocognitive tasks (e.g., Junttila et al., 2022) could be traced back to inherited factors in dogs (i.e., due to random or directional forces of selection). From the hypothetical proto-dog that initially joined our ancestors (see for various theories about the steps of early domestication, such as initial commensalism Dekel et al. (2017) or taming of ancient wolf pups Serpell (2021)) along the following millennia several more-or-less task-specific working dog types were formed via artificial selection (e.g., Ostrander et al., 2017). Towards the nineteenth century these provided the basis for the standardization of modern dog breeds (Pemberton et al., 2018). Tasks in which humans involve dogs include a wide range of activities such as (hunting, herding, protecting and guarding, being a draft animal or even to serve as a lapdog, among others). Performing well in these roles most likely requires different sociocognitive skills regarding the dog–human interactions as well (Pongrácz & Dobos, 2025a). Among the likely candidates for these ‘task-selected’ cognitive capacities we can mention persistence/inhibition (Hall, 2017), interspecific communication (Gnanadesikan et al., 2020), and observational learning (Dobos & Pongrácz, 2023, 2024). The investigation of breed- or breed group-related differences in dogs’ engagement and performance in interactive contexts with humans could boost our understanding of the post-domestication evolution of the sociocognitive capacities of dogs.

Various non-genetic factors (e.g., age: Chapagain et al., 2020; training: Marshall-Pescini et al., 2016; exposure to humans: Zaine et al., 2015) can affect the performance of dogs in a number of interactive tasks with humans. However, there are also indications for genetically influenced differences in the sociocognitive features of dogs. Bray et al. (2021) found that over 40% of the variation in point-following capacity, and gazing towards human faces, can be attributed to genetic factors in 8-week-old retriever puppies. It was also found that the separation of some breeds to ‘show’ and ‘working’ lines resulted in detectable differences in their problem-solving capacity (Fadel et al., 2016). Finally, there are many publications that found breed-related differences in various sociocognitive behavioural traits (see for a review Pongrácz & Dobos, 2025a).

From the functional and evolutionary aspects of ethological research (Shettleworth, 1974), it would be crucial to find an ecologically valid framework for the investigation of genetics-based behavioural differences in dogs. This means that in the case of ethological comparisons among dog breeds, such testing paradigm should be chosen where a given behavioural phenotype’s adaptive nature can be tested in a biologically meaningful way (Pongrácz & Dobos, 2025a). In the present study, the targeted canine behavioural phenotype was the tendency of paying attention to human ostensive communication, and we posed the question whether this tendency would be a function of the selection for creating dog breeds that differ in their working style with humans (‘cooperative’ versus ‘independent workers’).

Dogs show specific sensitivity to the qualities of human vocalizations, with both lexical content (known versus unknown words: Andics et al., 2016) and non-verbal features (signaller’s inner state: Andics et al., 2016; prosody, ‘dog-directed speech’: Gergely et al., 2021) being meaningful factors. Ostensive communication has unique lexical (words that address the receiver) and prosody-related characteristics (Wilson & Wharton, 2006). Specific sensitivity to human ostensive communication in human infants is a leading factor for rapid learning of behavioural rules and aiding observers in problem solving tasks (the so-called ‘natural pedagogy’ theory: Csibra & Gergely, 2009). According to experimental evidence and consequent theoretical considerations, dogs’ sensitivity to human ostension can be one of the important factors in the rapid absorption of learning rules (perseverative errors: Topál et al., 2009a; following human gazing signals: Duranton et al., 2017; Téglás et al., 2012) and to follow behavioural solutions observed from humans (reward quantity preference: Marshall-Pescini et al., 2012; spatial problem solving: Dobos & Pongrácz, 2024). Unlike the well-socialized companion cats (Pongrácz & Onofer, 2020), who showed no specific attraction to human actions accompanied with ostensive speech, it is assumed that socialized dogs show a general capacity for being attentive and sensitive to ostensive verbal signalling by humans (even from a young age: Byosiere et al., 2023). This capacity is assumed to provide them with an adaptive advantage to coexist and cooperate with humans, especially when (observational) learning opportunities arise (Pongrácz et al., 2004).

Although one could expect that sensitivity to human ostensive speech is more or less uniformly present in dogs, there is also accumulating empirical evidence that various sociocognitive traits can vary among ancestry clades (e.g., Pongrácz & Dobos, 2025b) and functionally different groups of dogs (e.g., Hecht et al., 2019). Dog breeds offer an obvious choice for the researcher to test clusters of dogs where one would expect within-group similarity and between-group differences in the targeted behavioural phenotype (Pongrácz & Dobos, 2025a). Recently new evidence has started to accumulate that post-domestication, directional selection, which aimed to develop dog breeds for different working purposes, could cause differences in various behavioural traits. Based on the genetic analysis of more than 2,000 mongrel and pure-bred dogs, Morrill et al. (2022) found that behaviours showing the strongest segregation belonged to clusters that characterize either the cooperative or the independent working dog types. The cooperative breeds had high scores on such genetically determined features as ‘biddability’ and ‘easy to train’. At the same time, single individual breeds could not be characterized with ‘breed-typical’ behaviours in that study. Specific working tasks are also likely to overlap significantly with close genetic relatedness among the dog breeds (e.g., sled dogs; or most terriers cluster together within separate genetic clades: Parker et al., 2017). This can result in such differences between the breeds’ sociocognitive features that seemingly are the function of their ancestry (i.e., the breeds’ evolutionary distance from the wolf-like ancestor: Dutrow et al., 2022). However, to gain more insight into the specific forces of selection that shaped the sociocognitive performance of dogs, it would be more advantageous to find such overarching factors that could have been developing independently from the degree of relatedness between dog breeds.

When interactions with humans are the focus of interest, an especially promising clustering feature of dog breeds emerges: their cooperative or independent working style with humans. Independent breeds have been selected for tasks in which they have to work either out of sight of their handlers, or without being regularly instructed (such breeds are, for example, the various terriers, sled dogs, and greyhounds). Cooperative breeds, on the other hand, have been selected for tasks where they work under close human supervision with regular feedback from the handler (such as herding dogs, gundogs, police dogs: Dobos & Pongrácz, 2023; Gácsi et al., 2009). It was found that cooperative dogs not only follow human visual communicative signals more successfully (Gácsi et al., 2009), but when several representatives of the two breed groups were tested in the detour paradigm, the independent working dogs did not benefit from observing a human demonstrator’s action (Dobos & Pongrácz, 2023). It is an intriguing question: what can cause the difference between the two breed groups’ performance in a communicative or social learning scenario? In another study, independent and cooperative breeds were again tested in the detour paradigm, where the experimenter was using either ostensive or neutral tone verbal signals during the demonstration (Dobos & Pongrácz, 2024). It was found that cooperative dogs learned from the demonstrator in both conditions; however, the independent dogs’ performance did not improve, and they did not follow the demonstrator with their gaze either. This result suggests that functional breed selection could affect the dogs’ sensitivity either towards ostensive intonation or verbal communication in general.

In our present research we focused on the effect of ostensive intonation of verbal utterances on cooperative and independent dogs’ behaviour, but now in the classical perseverative error paradigm. Throughout five consecutive trials, the subjects had to find the reward behind one of the two hiding places. The experimenter conspicuously hid the reward before each trial, where the order of hiding events was A-A-B-B-A, to test whether dogs from the two functionally different working breed types would rely differently on ostensive speech. One experimental condition contained ostensive verbal cueing (ostensive intonation and calling the dog’s name while encouraging them to watch the experimenter) in which the experimenter showed the task to the dogs, while in the other condition, the experimenter recited a short poem in a neutral (non-ostensive) tone. As eye contact was found to be an important factor in dog–human communication (e.g., Kaminski et al., 2012), and here we wanted to investigate the role of ostensive and neutral speech on dogs’ behaviour, we kept the visual signalling identical in the two conditions (see the details in the General Procedure).

### Hypotheses and predictions

It has been shown that ostensive cues can achieve their effect through either (i) signalling the demonstrator’s intent for communication (e.g., Senju & Csibra, 2008) or (ii) simply directing the observer’s attention to the action of the demonstrator (e.g., Kano et al., 2018). The A-not-B paradigm is especially useful in differentiating between these two mechanisms. We expected that if dogs were affected by the attention-enhancing effect of ostension, they would be more likely to correctly find the hidden reward in both hiding places. However, if ostensive cues affect dogs through signifying the demonstrator’s intention to share information with them (‘teaching a rule where to search’) instead, we expected that the dogs would develop a perseverative error pattern (look for the reward where it was found previously). We also hypothesized that the selection for cooperative or independent working style in the various breeds could have an additional effect on the dogs’ reliance on ostensive communication. We summarize our hypotheses and predictions in Table 1.

**Table 1.** **Hypotheses.** The first column shows the hypotheses we formulated for the study

	Neutral speech		Ostensive	
Hypothesis	Independent	Cooperative	Independent	Cooperative
Cooperative and independent working breeds are similarly sensitive to human’s ostensive communication	No perseveration: Topál et al. (2009a)*	No perseveration: Topál et al. (2009a)*	Perseveration: Topál et al. (2009a)	Perseveration :Topál et al. (2009a)
Cooperative and independent working breeds show different sensitivity to human’s *ostensive communication*	No perseveration: Topál et al. (2009a)*	No perseveration: Topál et al. (2009a)*	No perseveration: Dobos and Pongrácz (2024)	Perseveration: Topál *et al*. (2009a); Dobos and Pongrácz (2024)
Cooperative and independent working breeds show different sensitivity to human *verbal communication*	No perseveration: Topál et al. (2009a); Dobos and Pongrácz (2024)	Perseveration: Dobos and Pongrácz (2024)	No perseveration: Dobos and Pongrácz (2024)	Perseveration: Topál et al. (2009a); Dobos and Pongrácz (2024)

* The experimental conditions of the test groups are shown in the upper two rows. The other cells of the matrix indicate the predictions for the given group according to the hypothesis. Here, ‘Perseveration’ and ‘No perseveration’ refers to the dogs’ behaviour in the third Trial (first ‘B-trial’), where ‘No perseveration’ means that the dog first looks for the reward at location ‘B’. ‘Perseveration’ means that the dog commits the perseverative error by first looking for the reward at location ‘A’. We also added supporting references to the predictions. Topál et al. (2009a) did not use neutral speech, only artificial noise in the non-ostensive condition of their test.

## Methods

### Ethics approval and consent to participate

The authors assert that all procedures contributing to this work comply with the ethical standards of the relevant national and institutional guides on the care and use of animal subjects. The testing protocol was reviewed and approved by the Institutional Animal Welfare Ethics Committee, under the licence number ELTE-AWC-010/2023. The full procedure was performed in accordance with the Guidelines for the use of animals in research described by the Association for the Study Animal Behaviour. The tests were fully reward-based and non-invasive. The dog owners were present all along, assisting the experimenter. Informed consent was obtained from the dog owners before involving their dogs in the study. Before the tests, dog owners were informed about the procedure, and that they could stop the experiment any time if they thought that their dog experienced too high stress (this has never happened).

### Dog participants

Our subjects were minimum 1-year-old companion dogs who belonged either to the visually cooperative working breeds or to the visually independent working breeds. The work function of the breeds was determined based on the original breed standards. We did not test breed hybrids (e.g., Labradoodles) and we also avoided testing non-working breeds (‘toy’ or ‘lapdogs’, such as Bichon Bolognese, Pomeranian) and breeds where the original function has been entirely changed (e.g., English bulldogs). Table 2 shows the list of the successfully tested dogs and their basic details, as well as group assignments. To prevent overrepresentation of any breeds, we aimed to include no more than 3–4 subjects from the same breed to any of the experimental groups. We included the data in the statistical analysis of *N* = 20 cooperative dogs in the ‘ostensive speech’ group; *N* = 18 cooperative dogs in the ‘neutral speech’ group; *N* = 20 independent dogs in the ‘ostensive speech’ group; and *N* = 17 independent dogs in the ‘neutral speech’ group. Ten additional dogs had to be excluded from the experiment (thus their data were not entered into the analysis), because they were not motivated enough to perform the trials (the group assignment of excluded subjects was: 1 cooperative/ostensive; 3 cooperative/neutral; 4 independent/ostensive; 2 independent/neutral).

**Table 2.** List and details of the participating dogs whose data were included to the statistical analyses

Subject ID	Breed	Group	Demo	Sex	Reproductive status	Age (years)
1	Australian Shepherd	Cooperative	Ostensive	Male	Intact	3
2	Australian Shepherd	Cooperative	Ostensive	Male	Neutered	4
3	Border Collie	Cooperative	Ostensive	Male	Neutered	2.5
4	Border Collie	Cooperative	Ostensive	Male	Intact	1
5	Border Collie	Cooperative	Ostensive	Female	Spayed	10
6	Border Collie	Cooperative	Ostensive	Male	Neutered	4
7	Dutch Shepherd	Cooperative	Ostensive	Female	Spayed	7
8	Springer Spaniel	Cooperative	Ostensive	Female	Intact	1
9	Springer Spaniel	Cooperative	Ostensive	Female	Intact	4.5
10	German Shepherd Dog	Cooperative	Ostensive	Male	Intact	10
11	German Shepherd Dog	Cooperative	Ostensive	Male	Intact	1.5
12	Golden Retriever	Cooperative	Ostensive	Female	Spayed	1.5
13	Golden Retriever	Cooperative	Ostensive	Female	Intact	4
14	Irish Setter	Cooperative	Ostensive	Male	Neutered	11
15	Labrador Retriever	Cooperative	Ostensive	Female	Intact	2
16	Lagotto Romagnolo	Cooperative	Ostensive	Female	Intact	1.5
17	Lagotto Romagnolo	Cooperative	Ostensive	Male	Neutered	7
18	Lagotto Romagnolo	Cooperative	Ostensive	Male	Neutered	4
19	Malinois	Cooperative	Ostensive	Female	Intact	2
20	Mudi	Cooperative	Ostensive	Male	Neutered	1
21	Australian Shepherd	Cooperative	Neutral	Female	Spayed	2.5
22	Australian Shepherd	Cooperative	Neutral	Female	Intact	7
23	Australian Shepherd	Cooperative	Neutral	Female	Spayed	3
24	Border Collie	Cooperative	Neutral	Female	Spayed	2.5
25	Border Collie	Cooperative	Neutral	Male	Neutered	2
26	Border Collie	Cooperative	Neutral	Male	Intact	1.5
27	Border Collie	Cooperative	Neutral	Female	Spayed	6
28	English Cocker Spaniel	Cooperative	Neutral	Female	Spayed	2
29	English Cocker Spaniel	Cooperative	Neutral	Female	Spayed	1
30	Pointer	Cooperative	Neutral	Female	Spayed	8
31	German Shepherd Dog	Cooperative	Neutral	Female	Spayed	8
32	Golden Retriever	Cooperative	Neutral	Male	Neutered	3
33	Irish Setter	Cooperative	Neutral	Female	Intact	5.5
34	Labrador Retriever	Cooperative	Neutral	Male	Intact	2
35	Labrador Retriever	Cooperative	Neutral	Male	Neutered	6
36	Lagorai Shepherd	Cooperative	Neutral	Male	Neutered	3
37	Puli	Cooperative	Neutral	Female	Spayed	9
38	Sheltie	Cooperative	Neutral	Male	Neutered	3
39	Akita Inu	Independent	Ostensive	Female	Spayed	4
40	Basset Hound	Independent	Ostensive	Female	Spayed	2
41	Borzoi	Independent	Ostensive	Male	Unknown	2
42	Borzoi	Independent	Ostensive	Male	Intact	2.5
43	Boxer	Independent	Ostensive	Male	Neutered	2
44	Dachshund	Independent	Ostensive	Male	Neutered	5
45	Dachshund	Independent	Ostensive	Female	Spayed	11
46	Dachshund	Independent	Ostensive	Female	Spayed	4
47	Hungarian Greyhound	Independent	Ostensive	Female	Intact	3
48	Hungarian Greyhound	Independent	Ostensive	Male	Intact	3.5
49	Hungarian Greyhound	Independent	Ostensive	Female	Intact	2.5
50	Hungarian Greyhound	Independent	Ostensive	Female	Intact	1.5
51	Irish Terrier	Independent	Ostensive	Female	Spayed	9.5
52	Irish Terrier	Independent	Ostensive	Female	Intact	3
53	Irish Terrier	Independent	Ostensive	Male	Intact	3.5
54	Komondor	Independent	Ostensive	Female	Intact	5
55	Parson Russell Terrier	Independent	Ostensive	Male	Neutered	10
56	Samoyed	Independent	Ostensive	Female	Intact	4
57	Shiba Inu	Independent	Ostensive	Male	Neutered	3
58	Transylvanian Hound	Independent	Ostensive	Male	Neutered	4
59	Airedale Terrier	Independent	Neutral	Male	Neutered	10
60	Akita Inu	Independent	Neutral	Female	Spayed	2
61	Basset Hound	Independent	Neutral	Female	Spayed	2.5
62	Borzoi	Independent	Neutral	Female	Intact	2
63	Greyhound	Independent	Neutral	Male	Neutered	2.5
64	Ibizan Hound	Independent	Neutral	Female	Spayed	4
65	Irish Terrier	Independent	Neutral	Male	Neutered	2
66	Jack Russell Terrier	Independent	Neutral	Male	Intact	4.5
67	Rhodesian Ridgeback	Independent	Neutral	Male	Intact	4
68	Samoyed	Independent	Neutral	Male	Intact	1
69	Shar Pei	Independent	Neutral	Female	Spayed	2.5
70	Shiba Inu	Independent	Neutral	Male	Neutered	7
71	Staffordshire Terrier	Independent	Neutral	Male	Intact	1
72	Welsh Terrier	Independent	Neutral	Male	Neutered	11
73	Welsh Terrier	Independent	Neutral	Female	Intact	2.5
74	Whippet	Independent	Neutral	Female	Spayed	5
75	Whippet	Independent	Neutral	Male	Neutered	3

No more than four subjects per dog breed were included in an experimental group; however, it should be noted that a full balancing of the breeds, and other signalment variables, was not possible between the groups.

Besides the sex and reproductive status, we have also recorded the housing conditions of the subjects (indoor-only or indoor–outdoor); and their training history (none; owner trains at home; one course at dog school; regular dog school; private trainer; work-/sport-specific training).

### General procedure

A video clip, showing the testing procedure, can be accessed through this link at FigShare: https://doi.org/10.6084/m9.figshare.30460595.

Upon their arrival, dog owners (O) gave their written informed consent, and the experimenter (E) explained to the O what to do, and what not to do, during the test. We asked the O whether the dog is motivated best with food or a favourite toy, and henceforth we used the reward selected by the O during the test. This way we could minimize the chance that some of our subjects would be under- while others over-motivated, which is likely to happen when only one type of reward is allowed in an experiment. We tested dogs only if the O was confident to let the dog off-lead and could reliably recall the dog at any time. The owners were informed about this criterion during the recruiting process; thus, we did not need to exclude any subjects because of difficulties with being off-lead at a public outdoor area. In the case of any doubt about safe recallability, the dog would not be tested. In general, the subjects were familiar with supervised outdoor walks in city parks (such as where the testing site was) and many of them have visited the university before. However, the exact location where these tests were run was previously unknown to them.

All tests were run outdoors on a flat, grassy area at the campus of the Eötvös Loránd University (Budapest, Hungary). This area was far from any road and provided a secluded place for testing, without any disturbance of trespassing people. Each dog was tested only once. All tests were conducted between September and November 2023, during daylight hours. Weather conditions have been taken into consideration. We did not run tests in rain and avoided testing in the hottest part of the day in early autumn. Near the testing site a large tree provided shade for the participants. The whole testing procedure took no more than 10 min. We tested all dogs in the so-called A-not-B error paradigm (Péter et al., 2015). We opted for this five-trial version of the perseverative error test instead of the 7-trial variant used by Topál et al. (2009a), because the method of Péter et al. (2015) avoids all procedural asymmetries between the A- and B-hiding events (except the fundamental and desired order-effect). The test involved a repeated hide-and-seek task, where the experimenter performed five consecutive, visible reward hidings behind one of two hiding places. The hiding place was constructed from a small plastic opaque screen (40 × 40 cm), standing in front of a plastic bowl (15 cm tall, 25 cm diameter). The reward was always placed into the bowl, so the dog could not see it unless it went behind the screen and approached the bowl close enough to be able to look into it from above. The distance between the two hiding places was 2 m, and the dog was released from a starting point that was 10 m away from each of the hiding places, with the dog positioned on the midline. Based on the order of the hiding events, the two hiding places were called A and B, the hiding order was A-A-B-B-A. The first hiding place (either left or right) was chosen randomly in every experiment. A video camera (BLOW Go Pro4U) attached on a tripod was placed on the midline 2 m behind the hiding places, facing the starting point.

At the beginning of each trial, we asked the O to hold the dog by its collar or harness at the starting point, facing towards the hiding places. The E carried the reward (food or toy) conspicuously in her hand straight towards the chosen hiding place, then went behind the screen from outside towards the middle and visibly placed the reward behind it (i.e., into the bowl). After the E placed the reward into the bowl, she showed her empty hands towards the dog. Then the E returned to the dog who was kept at the starting point by their O, without approaching the other location. The E behaved the same way in both conditions, except for the intonation of her verbal signals (ostensive versus neutral speech). While the E walked towards the hiding places, she was facing away from the dog, but from time to time she looked back towards the dog and waved the reward in her hand. When she arrived at the hiding place, she turned towards the dog and looked at it. Then, she also kept her eyes on the dog while she walked back towards the starting point, where the O and the dog were waiting. After the E returned to the starting point, the dog was released, and it was allowed to go and find the reward.

The maximum length of a trial was one minute; if the dog did not find the reward during this time, it was recalled to the starting point and the next trial started. If the dogs did not choose in the first or second trial, the trial was repeated; therefore, every dog had two completed ‘A’ trials before hiding the reward to place B. Here, ‘completed’ means that the dog found the reward, but not necessarily by choosing the correct location first. Importantly, the dog was always allowed to find the reward even if it first went to the ‘empty’ location.

We had two experimental conditions. In the *ostensive condition*, in each trial, the experimenter maintained the dog’s interest (by calling their name and using attention-grabbing words such as ‘Watch me’ in an attention eliciting tone) during the entire hiding event, including when she approached the screen and when she returned to the dog. The attention-eliciting phrases were uttered in a high-pitched, lively and friendly tone, similarly to the intonation used with children. There was approximately 1 s pause between two phrases. In the *neutral speech condition*, during the entire hiding event, the experimenter recited a short poem without any ostensive tone.

### Statistical analysis

We performed the statistical analysis with IBM SPSS.29 software. For coding the videos, the BORIS software (© Olivier Friard and Marco Gamba) was used. The coded behavioural elements are listed and described in Table 3. The raw data can be accessed at Mendeley Data (https://data.mendeley.com/preview/h2n74cnmsz?a=009b4faa-a2fd-486e-8e70-4b9c5621d225).

**Table 3.** Behavioural variables used for statistical analysis, and their description

Behavioural variable	Unit	Description
Success	Yes/no	The dog on the first attempt chooses the hiding place that contains the reward.
Choice latency	Latency (s)	The latency of reaching the first chosen bowl – the dog puts its nose behind one of the screens.
Initial direction	‘correct’; ‘middle’; ‘wrong’	The dog’s direction when it starts moving towards the hiding places. Correct: straight route towards the reward; Middle: middle route between the two screens; Wrong: opposite route leading towards the empty location.
Side-switching	Frequency (1/s)	The number of side-switching events between the wrong and the correct route divided by the latency of first choice.
Looking away	Relative duration	During the hiding event the dog does not look at the demonstrator. Overall duration of looking away divided by the duration of the demonstration.

GEE (generalized estimating equations) with binary logistics was used for comparing the success rates of dogs both within and between the experimental groups. We considered a trial as ‘successful’ if the dog visited the location where the experimenter hid the reward first. Trial (as repeated factor), experimental group, dog’s sex, housing conditions and training level served as independent variables. Dog’s age, as a continuous variable, was also added to the model. We also included biologically meaningful two-way interactions to the model (group with trial, housing and training, respectively). We applied backward model selection, and the results of the final model are reported.

Binomial tests with the Holm–Bonferroni correction were used to analyse whether dogs performed above, at, or below chance level (0.5) in the individual trials. Here we report the adjusted *p*-values for each occasion.

GEE with linear logistics was used for the analyses of the frequency of side-switching, relative duration of looking away, and first choice latencies. GEE with ordinal logistics was used for the analysis of initial direction. Trial (as repeated factor), experimental group, success in the given trial, housing conditions and training level served as independent variables. Dog’s age, as a continuous variable, was added to the model where we analysed the duration of looking away. We included biologically meaningful two-way interactions to the model as well (group with trial, success, housing and training, respectively; and trial with success). We applied backward model selection, and the results of the final models are reported.

To ensure interobserver reliability, a second coder assessed the video footages of 15 subjects (20%), and we calculated the Cronbach’s alpha values for the frequency variables. The coding proved to be reliable in the case of each: first choice latency (Cronbach’s alpha = 0.935), side-switching (Cronbach’s alpha = 0.864), and looking away (Cronbach’s alpha = 0.939). In the case of dog’s success and initial direction, the two coders had 100% agreement in the coding.

## Results

**Figure 1.**
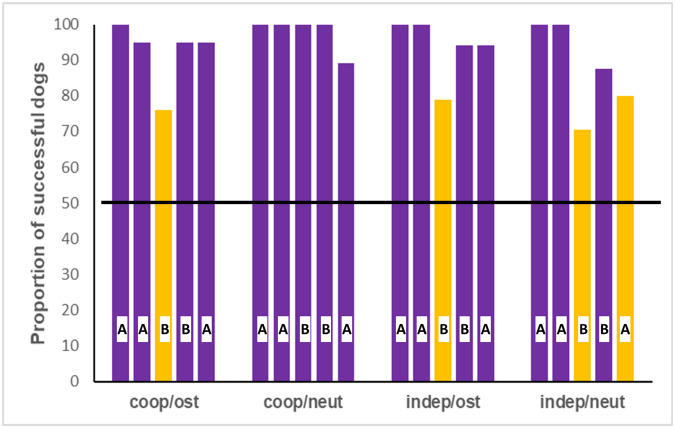
**Success rate of dogs in the four experimental groups**. The bars represent the trials each dog participated in. The capital letters ‘A’ and ‘B’ show the trial designation according to the first and second hiding place. Horizontal black line shows the 50% chance level. Purple bars indicate performance significantly above chance level, and gold bars indicate trials where success rate dropped to chance level (binomial tests with Holm–Bonferroni correction).

First, we analysed whether the success rate of dogs differed from the chance level in the individual trials (Table 4, Fig. 1). Cooperative dogs in the neutral speech group found the reward above chance level in each trial. In the other three groups, the success rate dropped to chance level in Trial 3 (first ‘B’ trial), and additionally, independent dogs in the neutral speech group could not exceed the chance level in Trial 5 either.

**Table 4.** Results of the binomial tests in the case of the success rates in the individual trials of each group

Group (*N*)	Trial	*P* (Holm–Bonferroni)
Cooperative breeds, ostensive speech, *N* = 21	1	0.02
	2	0.02
	**3**	**0.081**
	4	0.02
	5	0.02
Cooperative breeds, neutral speech, *N* = 18	1	0.02
	2	0.02
	3	0.02
	4	0.02
	5	0.02
Independent breeds, ostensive speech, *N* = 19	1	0.02
	2	0.02
	**3**	**0.076**
	4	0.02
	5	0.02
Independent breeds, neutral speech, *N* = 17	1	0.02
	2	0.02
	**3**	**0.143**
	4	0.02
	**5**	**0.081**

Chance level was 0.5. We used the Holm–Bonferroni correction to reduce the false discovery rate, and here we report the adjusted *p*-values. Trials and *p*-values where the success rate was at chance level are marked in bold. Compared to the other trials where dogs succeeded above chance level, these non-significant results show a larger proportion of subjects who committed the perseverative error.

**Figure 2.**
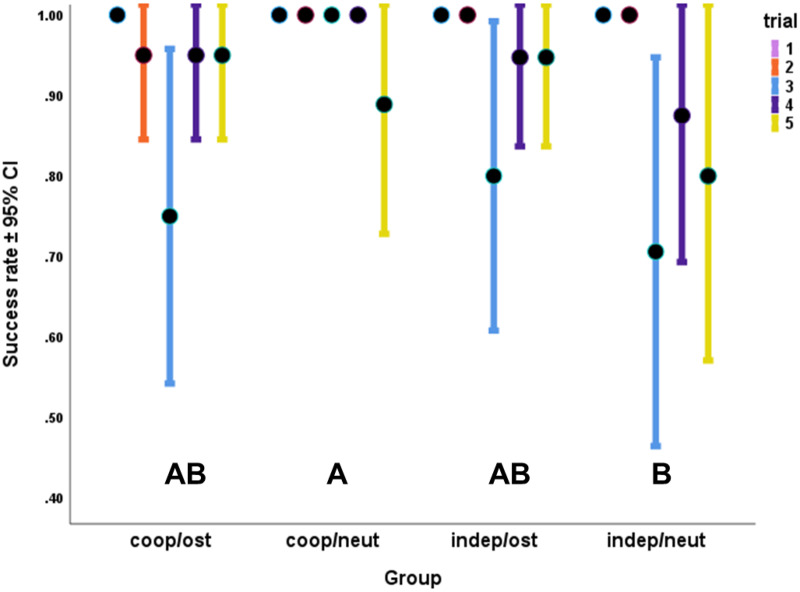
**The effect of the trials and testing groups** on the success rate. In the neutral speech condition, cooperative dogs were more successful than the independent dogs. Significant differences among the groups are indicated with different capital letters. The success rate was lower in Trial 3 than in Trials 1, 2 and 4 across all the groups. GEE with binary logistics.

When we compared the success rates across trials and between the experimental groups (GEE), we found significant association with the experimental group (Wald χ^2^_3_ = 15.352; *p* = 0.002), where cooperative dogs in the neutral speech condition were significantly more successful than independent dogs in the neutral speech condition (Fig. 2). Training also had a significant association with success (Wald χ^2^_5_ = 18.117; *p* = 0.003), but besides the main effect, we did not find significantly different groups according to the training level of dogs. Finally, trial also showed a significant association with dogs’ success (Wald χ^2^_3_ = 19.130; *p* < 0.001). Dogs were significantly less successful in Trial 3 than in Trials 1, 2 and 4 (Fig. 2). Dog’s sex (χ^2^_1_ = 0.320; *p* = 0.572), age (χ^2^_1_ = 0.261; *p* = 0.610), and housing conditions (χ^2^_1_ = 0.100; *p* = 0.752) did not have significant association with the success rate.

In the case of choice latencies, we only found a significant main effect of the trials (χ^2^_4_ = 26.491; *p <* 0.001). Here, dogs kept reaching the first chosen hiding place faster along the consecutive trials, with a significant difference between Trial 1 and Trial 4. First choice latencies did not show significant association with the experimental groups (χ^2^_3_ = 1.531; *p* = 0.675), success in the trial (χ^2^_1_ = 0.374; *p* = 0.541), housing conditions (χ^2^_1_ = 0.001; *p* = 0.982), or training level of the dogs (χ^2^_1_ = 0.965; *p* = 0.321).

The frequency of side-switching showed no significant association with any of the repeated or fixed variables: trials (χ^2^_4_ = 2.609; *p =* 0.625), experimental groups (χ^2^_3_ = 5.296; *p* = 0.151), success in the trial (χ^2^_1_ = 0.384; *p* = 0.536), housing conditions (χ^2^_1_ = 0.718; *p* = 0.397), and training level of the dogs (χ^2^_1_ = 0.258; *p* = 0.612).

**Figure 3.**
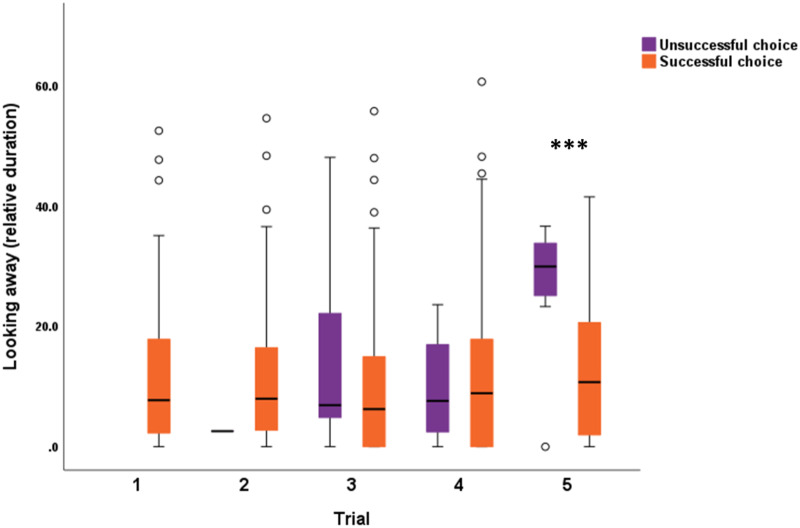
**The interaction between trials and success** in the case of the relative duration of looking away while the experimenter performed the hiding of the reward. In Trial 5, those dogs who looked away from the experimenter’s hiding action the most, remained unsuccessful. GEE with linear logistics. ***p < 0.001.

**Figure 4.**
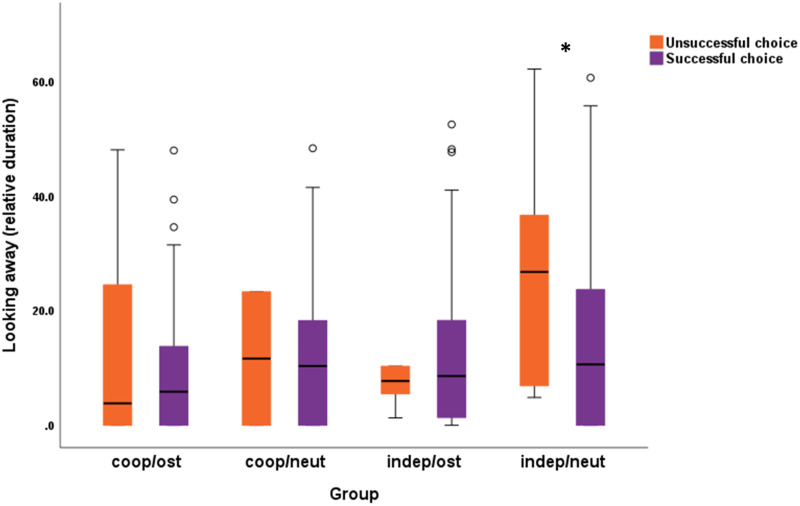
**The interaction between test group and success** in each trial in the case of the relative duration of looking away while the experimenter performed the hiding of the reward. Independent dogs in the neutral speech group looked away longer when they eventually erred in the trial. GEE with linear logistics. *p < 0.05.

In the case of relative duration of looking away (from watching the experimenter during the entire hiding event, between the experimenter’s departure from until her return to the starting point), we found two significant interactions: trials with success (χ^2^_3_ = 22.628; *p <* 0.001), and success with groups (χ^2^_3_ = 10.740; *p =* 0.013). In the case of the interaction between trials and the success in the given trial, dogs who spent less time looking at the experimenter in Trial 5, more likely erred (Fig. 3). The interaction between success and experimental group showed that independent dogs in the neutral speech group spent more time looking away from the experimenter when they erred, than when they were successful (Fig. 4). Dog’s age (χ^2^_1_ = 0.798; *p* = 0.372), housing conditions (χ^2^_1_ = 0.004; *p* = 0.947), and training level of the dogs (χ^2^_5_ = 3.688; *p* = 0.595) showed no significant association with looking away.

In the case of the initial direction, only the success showed a significant association (χ^2^_1_ = 28.080; *p* < 0.001). In most cases, dogs who erred in their choice started to go towards the ‘wrong’ hiding place at the beginning. Trials (χ^2^_4_ = 1.977; *p* = 0.740), experimental groups (χ^2^_3_ = 5.299; *p* = 0.151), housing conditions (χ^2^_1_ = 0.008; *p* = 0.930), and training level of the dogs (χ^2^_1_ = 1.363; *p* = 0.243) did not show significant association with initial direction.

## Discussion

We tested a wide assortment of working dog breeds in the A-not-B perseverative error paradigm. We found that both functional breed selection and the way the human experimenter spoke to the canine subjects during the hiding of the reward influenced dogs’ choice response in the critically important third trial. Cooperative breeds performed without any error in the neutral speech group, whereas the success rate of Trial 3 dropped to chance level in all the other groups. In other words, all dogs showed a higher rate of perseverative errors in Trial 3 in the case of ostensive verbal signals, and independent dogs in the neutral speech group also erred more frequently.

We can conclude that none of our initial hypotheses generated perfectly fitting predictions to the outcome of the experiment. Both breed types performed with decreased success rates in Trial 3 of the ostensive speech groups, which would fit with Hypothesis 1 (‘functional breed selection had no effect on dogs’ sensitivity to human ostensive intonation’). At the same time, Hypothesis 2 can be rejected because it predicted different performance in the breed groups when they received ostensive cueing. The higher rate of perseverative errors in both breed groups in Trial 3 also fits well to the earlier studies with dogs in the A-not-B paradigm (e.g., Péter et al., 2015; Sümegi et al., 2014; Topál et al., 2008), where dogs were almost indiscriminately sensitive to ostensive speech as the elicitor of perseverative errors. However, our first hypothesis also predicted that dogs would not commit the perseverative error in trials accompanied with neutral speech. This condition was an important addition to our test protocol, as previous research neglected the opportunity to compare the effect of ostensive and non-ostensive verbal signals on dogs in the A-not-B experiment, in which we actually found the strongest effect between the breed groups: while cooperative breeds reached the reward at its correct (‘B’) location each time in the neutral speech group, independent dogs showed the highest error rate among all groups in the third trial of the neutral speech group.

The difference between the performance of independent and cooperative dogs in the neutral speech groups was just the opposite compared to what we predicted in the case of Hypothesis 3. This hypothesis stated that functional breed selection could differently affect dogs’ general sensitivity to human communication. Earlier results regarding the following of visual pointing gestures (Gácsi et al., 2009) and learning from a human demonstrator in a detour task (Dobos & Pongrácz, 2023) inspired Hypothesis 3, as the independent breeds consistently underperformed the cooperative dogs in these human communication-based tasks. It is worth mentioning that newer results showed no difference between dogs’ performance in the human point-following task either connected to their breed or the ostensive/non-ostensive manner of the pointing (Espinosa et al., 2023). However, these authors used a more difficult-to-follow pointing technique than Gácsi et al. (2009) did (pointing with cross-lateral versus ipsilateral arm) and additionally sorted their subjects into more than two categories compared to the binary cooperative/independent categories used in the earlier study. These differences between the methods could cause the divergence of the results between the two studies on point-following. Additionally, recent research also highlighted that the independent breeds consistently paid less attention to the human demonstrator than cooperative breeds did in the detour task, regardless of the ostensive or neutral speech the demonstrator was using. Furthermore, the less the dogs watched the demonstrator’s action, the poorer their detour performance was (Dobos & Pongrácz, 2024). A similar explanation seems likely in our present A-not-B task, as independent dogs looked away the most from the experimenter’s walk while she was hiding the reward in the neutral speech condition. Furthermore, those independent dogs who watched the experimenter less in the neutral speech condition, more likely erred in Trials 3 and 5. In the case of independent dogs, neutral speech probably has a minimal to no attention-eliciting effect, and when they lose interest in the human’s action, they do not perform well in the detour task or in finding the reward in our current two-way choice task.

However, it was not only the independent dogs in the neutral speech group that erred more often in Trial 3; we found a similar pattern in both dog breed types in the ostensive speech groups. Earlier explanations for the higher incidence of perseverative errors in the first ‘B’ trial were based on the assumed effect of the experimenter’s repeated visits to location ‘A’ accompanied with ostensive speech. According to various publications (Péter et al., 2015; Topál et al., 2008) human infants may interpret this type of behaviour by the experimenter as a natural ‘teaching’ event, where the ‘lesson’ is the rule that the reward should be at location ‘A’. According to the explanation, dogs behaved in a functionally similar way as the infants: the ostensive, repeated hiding events to location ‘A’ elicited a fast rule learning in this domesticated species that is ready to follow behavioural templates shown by humans (Sümegi et al., 2014). It is tempting to assume that in our present investigation, both the independent and cooperative working dogs reacted with intense attention to the ostensively communicating experimenter and some of them quickly adopted a search pattern according to the ‘rule’: ‘*go to the “A” location*’. The results of the looking away time also support this explanation, as dogs in the ostensive groups only minimally turned their attention away from the experimenter. Moreover, this offers a plausible explanation for the frequency of errors by the independent dogs in the neutral speech group. Although the ostensive utterances elicited strong enough attention from both breed types, the neutral speech was probably too weak an attention-grabber for the independent dog breeds. This could cause confusion in some subjects, who expected stronger guidance from the experimenter. Or they simply did not pay attention to the experimenter and reverted to the ‘win–stay’ strategy (also reported by Péter et al., 2016). Interestingly, in the current A-not-B scenario, ‘rule learning’ reinforced by ostension or the ‘win–stay’ strategy elicited by more sporadic attention could result in functionally similar perseverative errors in Trial 3. The latter explanation seems especially plausible in Trial 5, in the case of the higher error rates of independent dogs with neutral intonation. We found here that dogs who did not pay close attention to the experimenter (longer ‘looking away’), erred more often, and this was mostly happening with the independent dogs.

Only the cooperative dogs in the neutral speech condition performed above-chance level in each trial. This result was correctly predicted by the first and second of our hypotheses, and it fits to the earlier investigations as well (e.g., Topál et al., 2009a). The difference between the breed groups (independent dogs committed perseverative errors more often in the neutral speech group) can be the result of the generally higher attention towards human activity and communication in the cooperative dogs. For them, neutral speech may be a strong enough attention grabber to be able to follow exactly where the experimenter hid the reward, but the lack of ostensive intonation resulted in no ‘rule learning’. Or, alternatively, the cooperative dogs may be able to quickly learn in this condition a ‘two-by-two pattern’ of hiding (A-A-B-B), thus they can easily predict that the fifth location would be an ‘A’ again. An interesting parallel can be drawn with the results of a recent detour/social learning research (Dobos & Pongrácz, 2024). There, cooperative dogs learned from the human demonstrator both in the ostensive and neutral speech groups. We can assume, that for the cooperative dogs, human verbal communication is a salient enough attention elicitor to follow the route of a walking human: they can learn an effective detour, and they can also find where the reward was hidden. However, to rapidly develop a searching rule in the A-not-B experiment (which is manifested in the higher rate of perseverative errors in Trial 3), they require ostensive speech.

An earlier study by Sümegi et al. (2014) showed that those dogs who were ‘overmotivated’ in toy retrieval games made less perseverative errors in the ostensive condition than dogs who showed average or low toy retrieval motivation levels. In our experiment the dogs’ motivation levels were likely not causing the differences we found between the breed types, as we always opted for the reward which, according to the owners’ experience, was the most motivating for the dog (food or toy); and we found the strongest difference between the breed types in the neutral speech condition. We should also be careful when comparing the results of Sümegi et al. (2014) to the present study’s outcome, as in that experiment the authors used a very different protocol (seven hiding events, where the experimenter at first always approached the ‘A’ hiding place). The result that training level had significant association with the dogs’ success rates could indicate that some types of training may elicit stronger attention from the dogs towards human activity or search tasks in general. However, as no significant differences were found between the individual training levels, we cannot be sure whether regular training could result in more effective learning of ‘rules’ from the dogs in this specific task.

The results of first-choice latency and initial direction showed that dogs became more confident (chose faster) along the consecutive trials; and they mostly started to walk towards that location where they subsequently checked for the reward first. This, together with the uniform occurrence of side-switching, confirms that when dogs eventually made an erroneous choice, this did not happen because they hesitated or had problems with the decision. Contrarily, they chose with similar confidence whether they made a good or an incorrect choice.

A limitation of this study could be that we performed the tests outdoors, and the outdoor conditions might influence cooperative and independent dogs differently. However, this could be equally true for an indoor environment. The experiment could be easily repeated among indoor conditions. In this case, if the testing room is not large enough to accommodate the measurements of our outdoor test (10 m distance between the starting point and hiding places), a shorter distance would most probably suffice. The use of non-uniform rewards (i.e., we opted for the type the owners considered the most motivating for their dogs) could theoretically result in different searching activities in the subjects who were looking for food versus a toy. However, if we had used the same reward for each dog, it would result in losing a considerable number of subjects because of lack of motivation.

## Conclusions

Functional breed selection in dogs offers a unique opportunity for the testing of such post-domestication events of artificial selection that directly affected human-directed behaviours and sociocognitive capacities in dogs (e.g., Bognár et al., 2021; Dobos & Pongrácz, 2023; Gácsi et al., 2009). This provides the chance for devising biologically relevant testing scenarios, where we can formulate ecologically valid hypotheses that investigate why particular behaviours would be adaptive for independent and cooperative working dogs in the anthropogenic environment (Pongrácz & Dobos, 2025a). The capacity for exceptionally fast rule-learning accompanied with their unique (‘human-like’) sensitivity to ostensive human communication is considered one of the hallmark capacities of dogs, enabling them to smoothly coexist and cooperate with humans (Topál et al., 2009a; Topál et al., 2009b). Our new results add important details to this view. While we found that independent and cooperative working dogs were probably equally capable of quickly developing ‘search rules’ if they observed an ostensively communicating experimenter, it was also shown for the first time that verbal utterances with neutral intonation had a very different effect on the two breed types. Whereas neutral speech was an effective enough attention-grabber for the cooperative dogs to result in a near-perfect success rate, the independent dogs were less keen to follow the action of the non-ostensively speaking demonstrator. Together with the findings of Andics et al. (2016), who found that familiar human words even with neutral intonation can be recognized by dogs, these results highlight the importance of investigating the effect of non-ostensive human speech on dogs.

The behavioural differences of independent and cooperative dog breeds in our current A-not-B search task and a recent detour experiment (Dobos & Pongrácz, 2024) provide interesting insight into the complexity of the effect of human verbal signals for dogs. Both the detour and the two-way search task can be considered spatial problem-solving. Although ostensive and neutral speech was an equally effective elicitor for social learning in cooperative dogs, they elicited markedly different behavioural responses from this type of dog breeds in the A-not-B task. This warrants that the exact nature of a task may require a different type of attention: for the cooperative dogs, neutral speech was a strong enough stimulus to observe the route of a human, while ostensive verbal cues may effectively elicit ‘rule learning’ about the potential patterns of human behaviour that eventually leads to perseveration.

Our results underline the importance of employing ecologically valid approaches in the investigation of dogs’ social cognition. Functional breed selection can provide an overarching background theory for breed-related behavioural differences, where we can develop biologically valid predictions for the adaptive responses of the different breed types, based on their specific pressures of selection in the past.

## Research transparency and reproducibility

All data generated or analysed during this study are included in this published article [and its supplementary information can be accessed at Mendeley Data (Pongrácz, Péter (2025), ‘A-not-B functional dogbreeds - Dobos_et_al2025,’ Mendeley Data, V2, doi: 10.17632/h2n74cnmsz.2)].
